# Prevalence and Determinant Factors of Intestinal Parasitic Infections and Undernutrition among Primary School Children in North-Central Ethiopia: A School-Based Cross-Sectional Study

**DOI:** 10.1155/2023/2256910

**Published:** 2023-03-15

**Authors:** Habtye Bisetegn, Habtu Debash, Hussen Ebrahim, Yonas Erkihun, Mihret Tilahun, Daniel Getacher Feleke

**Affiliations:** ^1^Department of Medical Laboratory Sciences, College of Medicine and Health Sciences, Wollo University, Dessie, Ethiopia; ^2^Department of Microbiology, Immunology and Parasitology, College of Health Sciences, Addis Ababa University, Addis Ababa, Ethiopia

## Abstract

**Background:**

Intestinal parasitic infections (IPIs) are a major public health problem with high morbidity and mortality in developing countries. Undernutrition is a major health problem among school children and affects their cognitive development, psychological development, motor skills, and academic achievements. Therefore, this study aimed to assess the prevalence and determinant factors of IPIs and undernutrition among primary school children.

**Method:**

Cross-sectional study was conducted among 450 children from February to March 2021 at selected primary schools in Dessie town, North-central Ethiopia. Participants were selected using a stratified sampling technique. Pretested questionnaires were used to collect sociodemographic and nutrition-related data. Stool samples were used to diagnose IPIs. Participants' height and weight were measured and body mass index (BMI) was calculated. Nutritional assessment was done using WHO AnthroPlus software. Data were analyzed using SPSS version 26 software. *P*-values <0.05 were considered statistically significant.

**Result:**

The overall prevalence of intestinal parasites was 28.9%. The prevalence of intestinal protozoa and helminths were 19.1% and 9.8%, respectively. *Entamoeba histolytica/dispar* was the most prevalent parasite (9.3%) followed by *Giardia intestinalis* (7.6%), *Enterobius vermicularis* (2.9%), and *Ascaris lumbricoides* (2.7%). The prevalence of intestinal parasites was higher in male (16.5%) than in female (12.4%) participants. Children whose mother's level of education is illiterate, 6–11 years old, have a habit of eating raw/undercooked fruits and vegetables, untrimmed and dirty fingernails, and sickness in the past week were significantly associated with IPIs. The prevalence of underweight, stunting, and wasting were 22.4%, 26.2%, and 20.7%, respectively. Multivariable logistic regression showed gender, family size, meal frequency, and breakfast were significantly associated with undernutrition. IPIs had a statistically significant association with underweight, stunting, and wasting.

**Conclusion:**

The study showed that IPIs and undernutrition are still major health problems among children in North-central Ethiopia. Periodic deworming, community health, and school health education will be valuable to improve the health, growth, and educational outcome of children.

## 1. Introduction

Intestinal parasitic infections (IPIs) are among the most common neglected tropical diseases (NTDs) that cause a major public health problem in developing countries. They are transmitted to human either directly through fecally contaminated food and water or indirectly due to poor environmental and personal hygiene [1, 2]. According to World Health Organization (WHO) technical report (1986), parasites affect more than 3.5 billion people and cause illness to 450 million people worldwide [3]. In the last decades, Sub-Saharan Africa and Asia contributed to the highest burden of IPIs [4].

Infections with intestinal protozoa such as *Entamoeba histolytica/dispar* and *Giardia intestinalis* and/or helminths such as *Ascaris lumbricoides*, *Trichuris trichiura*, hookworm, *Enterobius vermicularis*, and *Hymenolepis nana* are directly associated with low socioeconomic status, poor sanitation practices, contaminated water supply, lack of access to medical care, poor environmental conditions, and poor health status [5]. IPIs are more frequent among school children, because of their poor hygienic condition, typical hand-mouth activity, uncontrolled fecal activity, and immature immune system [6]. In school children, it is significantly associated with malnutrition, iron deficiency anemia, weight loss, low concentration, diarrhea, and stunted growth, which leads to impaired physical, intellectual, and cognitive development [7–9].

Malnutrition is the cellular imbalance between the supply of nutrients, energy, and the body's demand for them to ensure growth, maintenance, and specific functions [10]. In 2015, more than 795 million people in the world are malnourished, and the vast majority of undernourished people (780 million) continue to live in developing regions of the world [11]. This report showed a substantial decrease of 167 million undernourished people over the last decade, and 216 million less than that reported in 1990 [11]. However, in 2019, hunger was again on the rise with more than 820 million people being undernourished and more than 2 billion people in the world experiencing moderate to severe food insecurity underscoring the immense challenge of achieving zero hunger by 2030 [12]. Wasting and stunting negatively affect the cognitive communication, academic achievement, motor skills, and psychological development of children [13, 14]. More than 171 million children were stunted in Africa by 2010 with an estimated prevalence rate of 37.1% in 2020 [15]. Stunting and wasting are reported to be strongly associated with poor academic performance among school children [14].

A review conducted in 2010 about NTDs in Sub-Saharan Africa reported as Ethiopia is second to Nigeria to have the highest burden of IPIs in Sub-Saharan Africa. In Ethiopia, *A. lumbricoides*, *T. trichiura*, and hookworm affect 26, 21, and 11 million people, respectively. Ethiopia stands the second, fourth, and third to have the highest burden of ascariasis, trichuriasis, and hookworm infection in Sub-Saharan Africa, respectively [16]. A systematic review and meta-analysis reported 46% (95% CI: 38.50, 53.68) pooled prevalence of IPIs among primary school children in Ethiopia [17]. In studies conducted in different parts of Ethiopia, such as Birbir town [18], Sasiga district [19], Chencha town [20], and Bahir Dar town [21], the prevalence of IPIs was reported to be 27.1%, 62.4%, 81%, and 65.5%, respectively.

In Ethiopia, the prevalence of stunting ranges from 26.6% to 57.7%, wasting ranges from 3.2% to 16.7%, and underweight ranges from 11.9% to 24.5% [22]. A school-based cross-sectional study conducted in Northwest Ethiopia reported the prevalence of stunting, underweight, and wasting to be 27.5%, 20.4%, and 8.7%, respectively [23]. According to a systematic review and meta-analysis among school children in Ethiopia that analyzed 39 studies published between 2006 and 2019, the overall pooled prevalence of stunting, underweight, and wasting were found to be 21.3% (95% CI: 17.0, 25.5), 18.2% (95% CI: 14.4, 22.0), and 17.7% (95% CI: 13.5, 21.8), respectively [24].

The impacts of IPIs are not limited to morbidity and mortality but also extend to malnutrition. Screening of school children for IPIs and malnutrition and giving appropriate treatment are critical to avoid mortality and morbidity as well as to improve the physical, intellectual, and cognitive development of children. IPIs and undernutrition should be investigated in different localities, especially in low and middle-income countries, where poverty is the first issue. The outcome of the study will trigger/initiate the school community, district health office, healthcare workers, and other stakeholders who are working towards the prevention and control of IPIs and malnutrition. Based on the outcomes, either the exciting prevention and control strategies will be intensified or new strategies might be designed both in the community and in that specific primary school. Moreover, there are no comprehensive studies that evaluated the nutritional status of school children and factors associated with IPIs and undernutrition among school children in the study area. Therefore, this study aimed to provide documented evidence about the prevalence of IPIs and nutritional status and their determinant factors among primary school children in Dessie town, North-central Ethiopia. This was the first study in North-central Ethiopia to report the prevalence estimates for undernutrition among school children in the study area based on the WHO [25].

## 2. Materials and Methods

### 2.1. Study Area and Period

This study was carried out from February to March 2021 at selected primary schools in Dessie town. Dessie town is located in Amhara national regional state, South Wollo zone, North-central Ethiopia, about 400 km from Addis Ababa, the capital city of Ethiopia. It sits at a latitude and longitude of 11°8′;N 39°38′;E, with an elevation between 2,470 and 2,550 m above sea level. In the town, there are about 30 primary schools (grades 1–8). The town has a population of more than 200,000 people.

### 2.2. Study Design

A school-based cross-sectional study was conducted at four selected primary schools in Dessie town to determine the prevalence of IPIs, malnutrition, and associated factors among school children aged 6–17 years old.

### 2.3. Sample Size Determination and Sampling Procedure

The sample size of this study was computed using the single population proportion formula for a cross-sectional study [26]. By taking the prevalence of IPIs at 16% from a previous study conducted in Dessie town [27] and with the assumptions of 95% CI, 5% margin of error, and 10% nonresponse rate, the sample size was found to be 228. Then using a design effect of 2, the final sample size was calculated to be 456.

Thirty governmental schools, which are found in Dessie town, were registered and four primary schools were selected randomly. A stratified sampling method was used to select the study participants. Stratification was made based on the grade in which the students were attending and by sex to balance the male to female ratio. Then the number of students to be sampled was proportionally allocated to each school and grade based on the number of students in each school and grade. Based on this, a total of 120 students from Addis Fana primary school, 113 students from Dawdo primary school, 110 students from Etegie Menen primary school, and 107 students from Tossa primary school were included in the study. The study units were then selected by simple random sampling using class rosters as a sampling frame.

### 2.4. Eligibility Criteria

All registered students who are grade 1 to grade 8 and capable of providing a written informed assent form signed by their parents or guardian were included in this study. Children taking antiparasitic drugs in the past 4 weeks and nutritional supplements were excluded. Children who did not volunteer to participate were also excluded. In addition, children with other chronic diseases were excluded.

### 2.5. Data Collection Process

Sociodemographic characteristics, hygienic practices, environmental sanitation, and nutrition-related data were collected by pretested structured questionnaires that were prepared in English and translated to the local language Amharic. After data collection, the data was back translated to English for entry and analysis. The respondents were students and parents/guardians of the selected students. After students were systematically selected from the schools, their household address was traced in the students' parent database. Trained data collectors went to the children's houses to interview parents/guardians. The students were asked if they had sickness in the past 1 week (a feeling of gastrointestinal tract complaints such as abdominal pain, abdominal discomfort, constipation, diarrhea, and so on in the previous week).

### 2.6. Sample Collection and Laboratory Analysis

Each study participant was advised to bring about 2 g of fresh stool in a labeled, clean, dry, and leak-proof stool cup. Clear instruction was given to the student on how to bring the stool sample by the investigators. The stool sample was repeatedly collected up to three times if the first and the second samples were found to be negative for intestinal parasites at 24 hours intervals, and this will increase the chance of finding intestinal parasites. The diagnosis was made based on the identification of helminths ova, protozoan cysts, and trophozoite stage using a light field microscope. The direct saline wet mount was prepared immediately after reception of the stool sample and examined microscopically for the diagnosis of motile trophozoite stage of protozoan as well as other stages of intestinal parasites. Furthermore, the remaining sample was preserved in 10% formalin and transported to Wollo University laboratory to perform the formalin-ether concentration technique as described elsewhere [28]. The formalin-ether concentration technique will increase the parasite detection rate, especially when the parasite load in the patient is low. This is because the technique uses a large sample value, as well as the use of ether, which will digest fecal debris and fats. This makes the field clear and increases the chance of identifying parasites.

### 2.7. Nutritional Assessment

The study participant's body weight was measured by an electronic digital scale to the nearest of 0.1 kg, and height was measured by a meter to the nearest of 0.1 cm. Before measurement of weight and height, the children were told to remove shoes, hair clips, and braids. In addition, each child was positioned with the feet together flat on the ground, heels touching the back plate of the measuring instrument, legs straight, buttocks and scapula on the backboard, and arms loosely at their side. Body mass index (BMI) was calculated as the weight in a kilogram of the individual divided by the square of their height in meters. The participant's date of birth, age in a month, age in years, and edema status were recorded. Then, the z-score values for *weight-for-height*, *weight-for-age*, *height-for-age*, and BMI-for-age were calculated using WHO AnthroPlus software [29]. Underweight, stunting, and wasting were defined following the WHO standard [25, 30]. A child was classified as underweight, stunting, and wasting if his/her z-score of *weight-for-age, height-for-age*, and *weight-for-age* was lower than minus two standard deviations (−2SD), respectively.

### 2.8. Quality Control

The collected data were checked for its completeness and validity. All laboratory reagents were checked for their shelf life, presence of impurities, and storage conditions. Each sample was labeled with the student's identification card to minimize preanalytical errors. The parasitological tests were done following standard operating procedures to minimize errors during the analytical phase of the laboratory investigation. To control observer bias, each stool sample was examined by two laboratory scientists at Wollo University, and in the presence of inconsistent results, the results were checked by a senior laboratory professional who was blind to the first result and it was taken as the final result of the examination. All weight and height measurements were taken twice to ensure accuracy.

### 2.9. Statistical Analysis

The collected data were analyzed using IBM SPSS Statistics version 26 (IBM Corporation, NY, USA). The nutritional assessment was analyzed using the WHO's Anthro software version 3.2.2. The distribution of categorical variables was assessed using descriptive statistics and presented in tables. Frequencies were used to determine the prevalence of IPIs, underweight, stunting, and wasting. Binary logistic regression was carried out to identify predictor variables attributed to the prevalence of IPIs, underweight, stunting, and wasting. Variables with a *P*-value of ≤0.25 in the univariable logistic regression were entered into the multivariable logistic regression. *P*-value <0.05 at 95% CI was considered statistically significant.

### 2.10. Ethics and Consent Statement

Before conducting this research, ethical clearance was obtained from the Ethical Review Committee of the College of Medicine and Health Sciences, Wollo University. Permission letter to conduct the study was taken from the Dessie town education department and directors of each school. The children and their parents/guardians were told as participation was fully voluntary and had no impact on their education. The objective was clearly explained to the participants and their parents/guardians. Written signed assent was taken from each participant's parent or legal guardian. Confidentiality was maintained at each step of the study process. Children with IPIs and/or undernutrition were linked to the health institution for antiparasitic treatment and nutritional supplements.

## 3. Results

### 3.1. Sociodemographic and Hygienic Characteristics of the Study Participants

In this study, a total of 450 primary school children from grades 1 to 8 consisting of 231 males and 229 females who give complete information were included. The response rate of this study was 98.7%. The mean age (±SD) of the study participants was 10.85 (±2.54) years old with a minimum and maximum age of 6 and 17 years, respectively. The majority of the participants (59.3%) belong to the age group 6–11 years old. About 56.7% of the study participants were in grades 1–4 (Table 1). More than half of the study participants reported as they have a habit of eating raw/undercooked fruits and vegetables. Half of the study participants reported to have the habit of eating raw fruits and vegetables (Table 2).

### 3.2. Prevalence and Distribution of Intestinal Parasitic Infections among Primary School Children in Dessie Town, North-Central Ethiopia, 2021

A total of 450 school children from four primary schools were examined for IPIs. About 28.9% (130/450) of the study participants were infected with at least one intestinal parasite. The prevalence of IPIs was significantly higher among males (16.5%) than females (12.4%) (AOR = 1.83; 95% CI: 1.04–3.22; *P*-value <0.036). A significantly higher proportion of IPIs was observed among children in the age group 6–11 years old (AOR = 2.96; 95% CI: 1.19–7.4; *P*-value <0.02). Although the difference was not statistically significant (*P*-value >0.05), a higher prevalence of intestinal IPIs was detected in Addis Fana primary school (9.3%) and Dawdo primary school (7.1%) (Table 3). Students whose grade levels are from grades 1 to 4 contributed to a higher prevalence of IPIs (17.3% vs 11.6%) (Table 3).

Seven species of intestinal parasites were identified in this study. Intestinal protozoa were detected in 19.1% of the study participants, while intestinal helminths were detected in 9.8% (44/450) of the study participants. The most predominant intestinal parasites identified in this study were *E. histolytica/dispar* (9.3%), followed by *G. intestinalis* (7.6%) and *E. vermicularis* (2.9%). About 2.2% of the study participants had a double infection by *E. histolytica/dispar* and *G. intestinalis* (Table 4).

### 3.3. Factors Associated with the Prevalence of Intestinal Parasites

In the present study, significant associations were observed between IPIs and some explanatory variables (*P* < 0.05). A significantly high prevalence rate of IPIs was observed among children in the age group 6–11 years old (AOR = 2.96; 95% CI: 1.19–7.4). Similarly, school children whose mother's education was illiterate were 3.32 times more likely to be infected with intestinal parasites than children whose mother's education are college and above (AOR = 3.32; 95% CI: 1.43–7.71). Students who ate raw fruits and vegetables showed a significantly higher proportion of IPIs (AOR = 4.02; 95% CI: 2.28–8.44) compared to those who did not. Accordingly, students with a habit of open field defecation (AOR = 4.39; 95% CI: 2.28–8.44), whose fingers are dirty and untrimmed (AOR = 2.45; 95% CI: 1.42–4.23), who did not wash hands after using the toilet (AOR = 3.58; 95% CI: 1.64–7.82), and sickness in the past 1 week (AOR = 4.44; 95% CI: 2.61–7.55) were also more likely to be infected with IPIs (Table 5).

### 3.4. Nutritional Status of the Study Participants

In this study, a total of 450 primary school children were assessed for underweight, stunting, and wasting. The mean (SD) of height was 131.2 (12.8), and the mean (SD) of *height-for-age* z-score was −0.2 (1.3). Similarly, the mean (SD) of weight-BMI was 20.01 (4.8), and the mean (SD) of BMI-for-age z-score was 0.3 (1.5). The overall prevalence of underweight, stunting, and wasting were 22.4%, 26.2%, and 20.6%, respectively. In the multivariable analysis, the odds of undernutrition were significantly higher among children infected with intestinal parasites than noninfected children (*P* < 0.05). The prevalence of underweight and stunting was significantly higher among males than females (*P*-value <0.05). A high prevalence of underweight (6.7%) and wasting (6.7%) was found in Addis Fana primary school compared to the other schools. On the other hand, the prevalence of stunting was highest in Dawdo primary school (8.2%).

### 3.5. Factors Associated with Undernutrition among School Children

Variables with a *P*-value ≤0.25 in the univariable logistic regression were entered into multivariable logistic regression. The results showed that male children were 2.4 and 1.5 times more likely to be underweight and stunted than females, respectively. Similarly, children in the age group 12–17 years old were more likely to be underweight and stunted compared to those in the age group 6–11 years old. Moreover, children whose family size >8 (AOR = 3.39; 95% CI: 1.41–6.53), and who never ate breakfast (AOR = 3.28; 95% CI: 1.78–6.57) were more likely to be underweight compared to children whose family size is <5, and who always eat breakfast, respectively. In addition, children with IPIs were more likely to underweight (AOR = 3.21; 95% CI: 1.81–6.21), stunted (AOR = 3.75; 95% CI: 2.03–6.21), and wasted (AOR = 3.61; 95% CI: 2.21–6.21) compared to the studied school children without IPIs (Table 6).

## 4. Discussion

IPIs and undernutrition are continued to be major public health problems in Ethiopia, especially among children. The present study was conducted to assess the magnitude of IPIs and undernutrition and their determinant factors among primary school children in North-central Ethiopia.

In this study, the overall prevalence of IPIs among primary school children was 28.9%. This finding was higher than that reported previously in Ethiopia: in Dessie town (16%) [27]; Medebay Zana district, Southwest Tigray (17.7%) [31]; Gondar town, Northwest Ethiopia (22.7%) [32]; and Delo-Mena district, Southeastern Ethiopia (26.6%) [33]. This difference might be due to environmental and geographical variation and differences in the study period, environmental sanitation, hygienic practice, implementation of deworming, and poor supervision of the children playing area. Moreover, taking repeated stool samples could increase the chance of recovering intestinal parasites. This was essential to not miss some parasites that have the characteristics of irregular excretion of eggs.

On the other hand, the prevalence reported in the present study was lower than that reported in other areas in Ethiopia, including Bahir Dar (61.7%) [34]; Dona Berber primary school; Wukro town, Eastern Tigray (60.7%) [35]; Zegie Peninsula, Northwestern Ethiopia (69.1%) [28]; Sasiga district, Southwest Ethiopia (62.4%) [19]; Chencha town, Southern Ethiopia (81%) [20]; and Jawi town, Northwest Ethiopia (57.88%) [36]. In addition, the finding of this study was lower than that reported in other countries, such as in Khartoum, Sudan (30%) [37], Yemen (90%) [38], Nigeria (67.4%) [39], and Gabon (61%) [40]. The lower prevalence of IPIs in this study might be due to differences in personal hygiene, deworming activities, and different health-related activities such as health education, construction of toilets, and waste disposal pins as done by Wollo University graduating health science students during their community based and team training programs.

The prevalence of intestinal protozoan parasites was found to be 19.1%, while the prevalence of intestinal helminths was 9.7%. This finding was lower than a report in Babile town, Eastern Ethiopia, which found a 13.8% prevalence of intestinal helminths [41]. The predominant parasites detected in this study were *E. histolytica/dispar* with a rate of (9.3%), *G. intestinalis* (7.6%), and *E. vermicularis* (2.9%). The prevalence of *E. histolytica/dispar* and *G. intestinalis* indicates fecal contamination of foods and drinks and poor hygienic practice in the community. Moreover, a previous study also showed the presence of poor knowledge of water, sanitation, and hygiene conditions (WASH) in Dessie town [27]. *A. lumbricoides* was detected in 9.2% of the studied participants. The prevalence of *A. lumbricoides* in the study area can be associated with the feature of the egg of the parasite to resist harsh environmental conditions. Accordingly, *Taenia species* was detected in 2% of the studied primary school children. This can be associated with the habit of eating raw/undercooked meat reported by the study participants.

The prevalence of IPIs was significantly higher in males than females (16.5% vs 12.4%). This agreed with a study done in Iran that reported a higher prevalence of IPIs among males [2]. In the present study, the prevalence of IPIs was more common in young children (6–11 years) and decrease in older children (12–17 years). This was similar to that reported in Iran [2]. This might be because children 6–11 years old have little awareness about the hygiene and poor sanitation practice of young children, and improved hygienic conditions and behavioral changes among older children. In addition, children at 12–17 have more competent immunity than young children.

The present study also showed a significant association between the prevalence of IPIs and the mother's level of education. The mother's role in food preparation and protection of children is high. Therefore, the mother's awareness and sanitation practices affect the health of their child.

According to the results of this study, stunting is much more common than being underweight and wasting. This is in line with a study that assessed the global estimated prevalence of stunting from 1990 to 2020 [15].

In this study, the prevalence of underweight was 22.4%. This finding was higher than the findings of studies conducted in Southwest Ethiopia (14.2%) [42]; however, the finding was lower than previous studies conducted in the Kishoreganj district of Bangladesh (40.5%) [43].

About 26.2% of the study participants were stunted. This finding was in line with the finding of a systematic review and meta-analysis that reported a 23.1% (95% CI: 19.0–27.0) pooled prevalence of stunting among school children in Ethiopia [21]. However, this finding was higher than the report of a study done in Jimma zone, Southwest Ethiopia (24.1%) [42], Northeast Ethiopia (14.1%) [44], and Bahir Dar, Ethiopia (15.3%) [45]. However, this finding was lower than the reports of studies conducted in Gondar, Northwest Ethiopia (46.1%) [46], Arba Minch, Southern Ethiopia (41.9%) [47], and Southern Ethiopia (28%) [48].

According to this study, the prevalence of wasting was 20.7%, which agreed with a study done in rural areas of Southern Pakistan that found 21% of school children were wasted [49]. This was higher than the reports of previous studies in Gondar, Northwest Ethiopia (46.1%) [46], Jimma zone, Southwest Ethiopia (9.1%) [42], Arba Minch, Southern Ethiopia (8%) [47], and Southern Ethiopia (14%) [48]. The variability in the prevalence of underweight, stunting, and wasting between this study and previous studies might be due to differences in community awareness about nutrition, socioeconomic status of families, health service coverage, poverty, and differences in the prevalence of IPIs and in dietary diversity.

According to multivariable logistic regression, the odds of being underweight among males were twice compared with that of females. This was supported by different studies conducted in Ethiopia that found males are more likely to be underweight compared to females [21, 45]. However, this finding contradicts a study conducted in rural areas of Southern Pakistan [49]. The likelihood of being underweight, stunting, and wasting has a positive correlation with the age of the students. Children of 12–17 years old are more likely to be underweight, stunted, and wasted compared to children of 6–11 years old. This is in line with a study done in Bahir Dar, Ethiopia that reported children older than 11 years are 15 times more likely to be stunted, and Pakistan that showed older age 3.6 times more likely to be underweight [45, 50].

Children whose family size was more than eight were nearly two times underweight, nine times stunted, and one time wasted compared to children with a family size of less than four. The risk of being undernourished was higher among children who never ate breakfast and had two times or at least meal frequency per day. This finding agreed with a meta-analysis done in Ethiopia [21].

This study found a higher prevalence of undernutrition among intestinal parasite-infected school children. School children with IPIs were two times more likely to be underweight, stunted, and wasted compared to those without IPIs. This disagrees with a report in Northwest Ethiopia that found no association between IPIs and undernutrition [32]. The higher prevalence of undernutrition among children with IPIs might be because intestinal parasites impair nutrient utilization by the body and decrease the level of iron and zinc, which are the most essential micronutrients for human growth and development [51, 52].

## 5. Limitations

In this study, special diagnostic techniques required for the detection of some parasites such as *E. vermicularis* and *S. stercoralis* were not employed. This might affect the reported prevalence of these parasites. Moreover, modified acid-fast staining was not performed for the detection of intestinal coccidian parasites. In addition, due to resource limitations, we did not evaluate the micronutrient level of the children.

## 6. Conclusions

The overall prevalence of IPI among the studied primary school children in North-central Ethiopia was 28.9%. Sex, age, grade level, mother's level of education, father's level of education, practicing open field defecation, eating undercooked fruits and vegetables, and raw meat, and sickness in the past week were significant predictors of intestinal parasites among the studied children. This study showed that underweight, stunting, and wasting are major health problems among the studied children; thus, malnutrition is still a major concern among primary school children in Ethiopia. Malnourished children are at risk of different health problems such as poor cognitive development, poor motor skills, and being prone to opportunistic infection due to the weak immune system. Undernutrition also led to high school absenteeism and poor academic achievement. This indicates the need to design and implement a deworming and school health and nutrition program to control parasitic infections and malnutrition-associated morbidity and mortality among school children.

## Figures and Tables

**Table 1:** Sociodemographic characteristics of primary school children in Dessie town, North-central Ethiopia, 2021.

Characteristics		Frequency	Percent (%)
Sex	Male	231	51.3
	Female	219	48.7
Age group in years	6–11	267	59.3
	12–17	183	40.7
Family size	<5	158	35.1
	5–8	264	58.7
	>8	28	6.2
Mother's education	Illiterate	87	19.3
	Primary	81	18
	Secondary	168	37.3
	College and above	114	25.3
Father's education	Illiterate	44	9.8
	Primary	49	10.9
	Secondary	199	44.2
	College and above	158	35.1

**Table 2:** Hygienic practice and food habits of the study participant in Dessie town, North-central Ethiopia, 2021.

Characteristics		Frequency	Percent (%)
Hand washing before eating	Yes	404	89.8
	No	46	10.2
Hand washing after using toilet	Yes	398	88.4
	No	52	11.6
Presence of toilet	Yes	420	93.3
	No	30	6.3
Practice of open defecation	Yes	83	18.4
	No	367	81.6
Cleanness and trimming of fingernail	Yes	331	73.6
	No	119	23.4
Eating raw vegetables and fruits	Yes	243	54
	No	207	46
Eating raw meat	Yes	69	15.3
	No	381	84.7
Milk consumption	Never	107	23.8
	Sometimes	269	59.9
	Always	74	16.4
Having breakfast	Never	47	10.4
	Sometimes	131	29.1
	Always	272	60.4
Sickness in the past week	Yes	130	28.9
	No	320	71.1

Sickness in the past week: children with gastrointestinal tract complaints in the past 1 week.

**Table 3:** Prevalence of IPIs among primary school children in Dessie town, North-central Ethiopia, 2021.

Characteristics		Intestinal parasites		*P*-value
		Positive (%)	Negative (%)	
Sex	Male	74 (16.5)	157 (83.6)	0.036
	Female	56 (12.4)	163 (87.6)	
Age group in year	6–11	70 (15.6)	197 (84.4)	0.02
	12–17	60 (13.3)	123 (86.7)	
Name of primary school	Addis Fana	42 (9.3)	78 (17.3)	0.74
	Dawdo	32 (7.1)	81(18)	
	Etegie Menen	30 (6.7)	80 (17.8)	
	Tossa	26 (5.8)	81 (18)	

**Table 4:** Prevalence of identified parasite species among school children in Dessie town, North-central Ethiopia, 2021.

Intestinal parasites	Frequency	Percent (%)
*Entamoeba histolytica/dispar*	42	9.3
*Giardia intestinalis*	34	7.6
*Enterobius vermicularis*	13	2.9
*Ascaris lumbricoides*	12	2.7
*Taenia species*	9	2
*Hymenolepis nana*	6	1.3
*Hookworm*	4	0.9
Mixed (*E. histolytica* and *G. intestinal*)	10	2.2

**Table 5:** Factors associated with the prevalence of IPIs among primary school children in Dessie, North-central Ethiopia, 2021.

Characteristics		Number of examined	Positive (%)	COR (95% CI)	AOR (95% CI)
Sex	Male	231	74 (33)	1.37(0.91–2.07)	1.83 (1.04–3.22)
	Female	219	56 (25.6)	Reference	Reference
Age group	6–11	267	86 (32.2)	1.50 (0.98–2.3)	2.96 (1.19–7.4)
	12–17	183	44 (24)	Reference	Reference
Mother's education	Illiterate	87	46 (52.9)	2.92 (1.62–5.28)	3.32 (1.43–7.71)
	Primary	81	26 (32.1)	1.33 (0.71–2.79)	2.16 (0.89–5.24)
	Secondary	168	31 (18.5)	0.77 (0.43–1.37)	0.748 (0.34–1.66)
	College and above	114	27 (23.7)	Reference	Reference
Father's education	Illiterate	44	21 (47.8)	2.23 (1.15–4.32)	2.02 (0.78–5.23)
	Primary	49	23 (46.7)	2.29 (1.2–4.37)	1.22 (0.49–3.05)
	Secondary	199	48 (24.1)	1.21 (0.74–1.97)	1.45 (0.76–2.97)
	College and above	158	38 (24.1)	Reference	Reference
Hand washing after using a toilet	Yes	398	104 (26.1)	Reference	Reference
	No	52	26 (50)	4.19 (2.21–7.96)	3.58 (1.64–7.82)
Practice of open field defecation	Yes	83	48 (57.8)	5.46 (3.26–9.12)	4.39 (2.28–8.44)
	No	367	82 (23.3)	Reference	Reference
Cleanness and trimming of fingernail	Clean	331	75 (22.7)	Reference	Reference
	Dirty	119	55 (46.2)	2.12 (1.39–2.37)	2.45 (1.42–4.23)
Eating raw/undercooked vegetables and fruits	Yes	243	96 (39.5)	2.9 (1.87–4.59)	4.02 (2.28–7.09)
	No	207	34 (16.4)	Reference	Reference
Eating raw meat	Yes	69	33 (47.8)	2.32 (1.39–3.87)	1.87 (0.95–3.69)
	No	381	97 (25.5)	Reference	Reference
Sickness in the past 1 week	Yes	130	78 (60)	3.83 (2.5–5.88)	4.44 (2.61–7.55)
	No	320	52 (17.3)	Reference	Reference

**Table 6:** Factors associated with the prevalence of underweight, stunting, and wasting among primary school children in Dessie town, North-central Ethiopia, 2021.

Variables	Underweight		Stunting		Wasting	
	Underweight *n* (%)	AOR (95% CI)	Stunted *n* (%)	AOR (95% CI)	Wasted *n* (%)	AOR (95% CI)
Sex						
Male	58 (12.9)	2.41 (1.64–5.61)	63 (14)	1.47 (1.14–1.89)	42 (9.3)	0.78 (0.52–1.18)
Female	43 (9.5)	Reference	55 (12.2)	Reference	51 (11.4)	Reference
Age group in years						
6–11	38 (8.4)	Reference	46 (10.2)	Reference	45 (10)	Reference
12–17	63(14)	2.01 (1.23–4.23)	72 (16)	1.27 (1.04–1.66)	48 (10.7)	1.78 (1.21–2.57)
Family size						
<5	14 (3.1)	Reference	25 (5.6)	Reference	27 (6)	Reference
5–8	28 (6.2)	1.31 (0.68–6.21)	30 (6.7)	2.24 (1.23–3.38)	30 (6.7)	1.67 (1.14–2.48)
>8	59 (13.1)	3.39 (1.41–6.53)	63 (14)	10.2 (1.76–16.8)	34 (7.6)	2.24 (1.33–3.40)
Having breakfast						
Never	54 (12)	3.28 (1.78–6.57)	58 (12.9)	2.38 (1.24–4.34)	36 (8)	1.89 (1.14–3.61)
Sometimes	26 (5.8)	1.74 (1.01–3.05)	33 (7.3)	1.21 (0.97–1.64)	31 (6.9)	1.29 (0.92–1.76)
Always	21 (4.7)	Reference	27 (6)	Reference	24 (5.3)	Reference
Meal frequency per day						
Two times or less	58 (12.9)	2.99 (1.7–12.03)	48 (10.7)	2.34 (1.1–10.54)	34 (7.6)	3.14 (1.58–4.88)
Three times	24 (5.3)	1.58 (0.99–3.64)	38 (8.4)	1.36 (0.26–6.8)	33 (7.3)	1.07 (0.90–1.80)
>Three times	19 (4.2)	Reference	32 (7.1)	Reference	24 (5.3)	Reference
Intestinal parasitic infections						
Yes	76 (16.9)	3.21 (1.81–6.21)	81 (18)	3.75 (2.03–6.21)	60 (13.3)	3.61 (2.21–6.21)
No	25 (5.6)	Reference	37 (8.2)	Reference	31 (6.9)	Reference

## Data Availability

All the data set used to reach the conclusions drawn in the manuscript are available within the manuscript.

## Abbreviations

IPIs: Intestinal parasitic infections
NTDs: Neglected tropical diseases
WHO: World Health Organization.
